# Internal Auditory Meatus Stenosis - Case Report

**DOI:** 10.1016/S1808-8694(15)31110-1

**Published:** 2015-10-19

**Authors:** Ivair Massetto, Alfredo Rafael Dell'Aringa, José Carlos Nardi, Kazue Kobari, Lilian Anabel Freitas Brandão, Laura Beatriz Vieira Fernandes

**Affiliations:** 1 1st Year ENT Resident Physician - FAMEMA; 2 PhD, Professor, Head of the ENT Residency Program - FAMEMA; 3 M.Sc. Assistant Professor of Otorhinolaryngology - FAMEMA; 4 Specialist in ENT. Assistant physician ENT - FAMEMA; 5 3rd Year ENT Resident Physician - FAMEMA; 6 5th Year Medical Student - FAMEMA

**Keywords:** auditory, stenosis, internal, meatus

Internal auditory meatus stenosis is defined as a loss of 3mm or more in the vertical diameter of the internal acoustic meatus, or even as a meatus smaller than 2mm.^1^

Inner ear abnormalities may occur in about 20% of the cases of patients who have sensorineural hearing loss^3^ (Figure 1).

**Figure 1:**
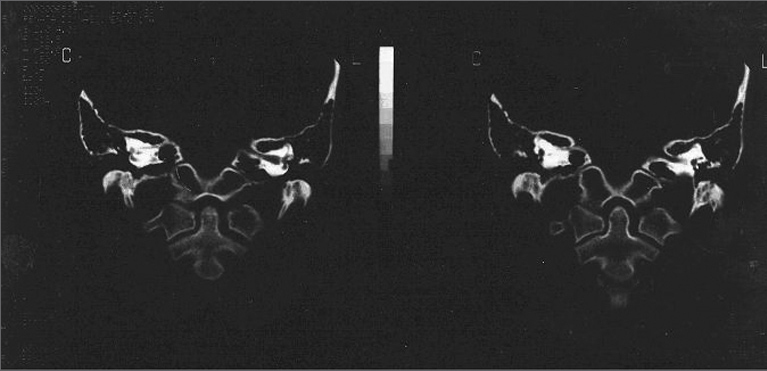
IAM stenosis on the right side.

This loss may happen because of alterations in the VIII cranial nerve (vestibulococlear).^2^

In most cases, it happens as an isolated congenital manifestation, and other systemic abnormalities are rarely found. The major alteration happens due to a constriction caused by impaired bone growth, resulting in an abnormal internal acoustic meatus. The stenosis etiology may be explained as being secondary to an aplasia or hypoplasia of the vestibulocochlear nerve. The embryologic events involved in fetal growth between the 4th and the 8th weeks are crucial for bone growth, and may cause such disease. The labyrinth may be also involved, being aplastic or with a deformed or incomplete cavity. It is not uncommon to have a completely normal labyrinth, though.^1^, ^2^, ^3^

An acquired bone disease may also result in stenosis of the internal acoustic meatus (osteomas, osteopetrosis, Paget's disease, and others).^1^

Clinical manifestations involve especially hypoacusis, there may also be tinnitus and vertigo in the side affected. When there is facial nerve involvement, there may be paresthesia and even paralysis.^3^

The physician should investigate the patient's obstetric, natal and post-natal history in order to rule out malformations during this period.^1^^,^^4^

Otorhinolaryngological exam involves otoscopy, which is usually normal, and also tests that investigate the patency of the VIII cranial nerve's vestibular branch (Romberg, Untemberg, heat tests, nystagmus and others).^3^

Among complementary tests, Audiometry may show a sensorineural hearing loss that varies as to the degree of hearing loss (from mild to profound), depending on the level of nerve involvement.

Diagnosis of certainty is made by CT scan, which shows a narrow acoustic meatus, thus pointing to the disorder. MRI can be used in order to see the structures that involve the VIII cranial nerve, which may be aplastic.^4^

As treatment modalities, the cochlear implant offers very promising results, as well as hearing aids, which help patients recover their hearing.

## CASE PRESENTATION

R.N., 18 years, female, came to our service complaining of right side hearing loss, noticed about one year ago, of unknown onset. She did not complain of tinnitus, vertigo, otalgia and otorrhea. She said she had not undergone any neonatal hearing screening test. Her hearing pattern did not get worse along the years. She also did not have learning disorders during childhood.

She did not show any evidence of pre-natal, natal and postnatal infection, nor of using ototoxic drugs.

She has essential hypertension and is being treated with propanolol (40mg) BID; nifedipine (20mg), administered once a day and hydrochlorothiazide also once a day.

She did not have relatives with hearing loss.

Her ear drums were intact and translucid. Her nose and throat exams were normal. Balance tests were also within the normal limits.

We followed the investigation protocol of hypoacusis in our service. We ordered: audiometry and immitanciometry, PEATE and OAE.

Her tests showed severe sensorineural hearing loss on the right side. PEATE did not show evidence of retrocochlear alterations.

Afterwards, we ordered ear CT scan and MRI, which showed stenosis of the internal acoustic meatus and vestibulocochlear nerve hypoplasia, respectively.

As treatment, we referred the patient to a hearing aid fitting, speech and hearing therapy and otorhinolaryngological follow up in our institution.
